# Velocity of Climate Change and the Vulnerability of Mountain Lake Landscapes

**DOI:** 10.1021/acs.est.5c03154

**Published:** 2025-08-15

**Authors:** Christine A. Parisek, Jonathan A. Walter, Steve Sadro, Andrew L. Rypel

**Affiliations:** † Department of Wildlife, Fish, & Conservation Biology, 8789 University of California Davis, Davis, California 95616, United States; ‡ Center for Watershed Sciences, University of California Davis, Davis, California 95616, United States; § Department of Environmental Sciences, University of Virginia, Charlottesville, Virginia 22903, United States; ∥ Department of Environmental Science & Policy, University of California Davis, Davis, California 95616, United States

**Keywords:** freshwater, mountain landscapes, high elevation lakes, climate change vulnerability, heat accumulation, velocity of climate change, speed of thermal change, growing degree days, killing degree days

## Abstract

Freshwater ecosystems in mountain landscapes are threatened by climate change. Accumulated heat can result in lethal short-term heat exposure, while velocity of change governs severity and rates of long-term heat exposure. Here, we novelly integrate heat accumulation and velocity of change approaches to classify climate-vulnerable USA mountain watersheds. We combine watershed position and air temperature data to calculate degree-days. We then calculate the current velocity of this change and used discriminant function analyses to classify watershed vulnerability through 2100. Our results demonstrate how rates of heat accumulation are increasing across mountain landscapes. We estimate 19% of watersheds are at greatest vulnerability to accumulated heat, and this will increase to 33% by 2100. Further, mean killing degree days (i.e., region-specific mean number of days above 90th temperature percentile) are projected to increase 215–254% (mean = 236%) over this same time frame. Together, results indicate heat accumulation will increase substantially over the next 75 years; changes are projected to be most severe in lower elevation landscapes and those with greatest historical velocity of change. These changes will likely restructure species’ distributions. Decision-makers can use these classifications to better understand landscapes, species’ needs, and ecosystem services, thereby enabling effective allocation of conservation resources.

## Graphical abstract

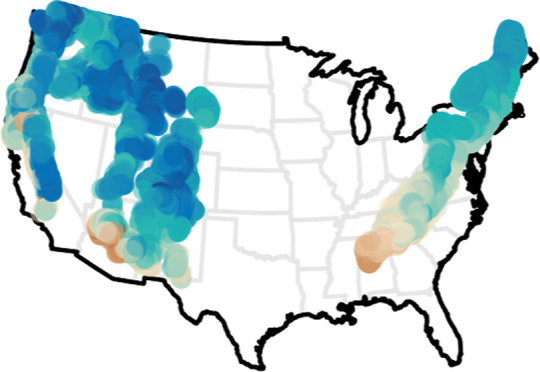

## Introduction

Rates of freshwater biodiversity loss outpace those of other environments, and protections for freshwater ecosystems are insufficient at almost all scales.^1–3^ Freshwater ecosystems are in global peril; human domination of the global water cycle undermines ecosystem stability and disrupts ecological organization.^4–6^ Climate change is desiccating wetlands, accelerating glacial retreat, and producing cascading consequences to ecosystem regimes, food web structure, and community functions.^7–9^ Indeed, impacts of climate change are triggering disruptions across all levels of organization in freshwater ecosystems.^5,7,10^ Climate-driven environmental disruption may be especially disruptive in mountain ecosystems, where terrestrial and freshwater taxa interact and often subsidize one another.^11–13^ Indeed, many mountain species, particularly fish, aquatic and terrestrial macroinvertebrates and arthropods, possess narrow thermal tolerances and restricted range distribution, thus climate adaptation via dispersal is highly limited.^14,15^

Temperature is perhaps the most important ecological variable mediating key ecological processes in aquatic ectothermic species.^16^ Understanding the role of temperature in regulating the distribution of organisms is therefore widely recognized as critical for understanding and managing freshwater biodiversity.^17–19^ Mountain landscapes are already thought to be exceptionally vulnerable to climate change.^20–22^ Therefore, quantifying heat accumulation and heat content of these areas is important.^23^ Nevertheless, nuance in how thermal regimes (i.e., the timing, magnitude, and velocity of temperature change or heat accumulation) holistically respond to climate change is important to quantify and understand.^24^ Short-term buildup of heat in aquatic ecosystems can lead to brief but lethal heat exposures, yet the velocity of this thermal change governs severity and long-term rate of exposure on the landscape. Velocity of change, in particular, is a useful indicator to understand not only the magnitude of climate change experienced by organisms, but also the quickening pace of that change.^25,26^ For example, high rates or degrees of change in ecosystems is associated with ecosystem fragility and abrupt shifts to alternate stable states.^27–29^

The consequences of potential increased velocity of climate change not only impacts aquatic ecosystems within catchments, but also the entire surrounding landscape.^30,31^ Kratz et al. (1997) described a lake’s position within a landscape as a combination of the spatial and ecohydrological contexts of the lake within larger lake districts.^150^ Climate-driven niche ranges of many mountain organisms are shifting upslope toward more suitable habitat, such as those of alpine grouse and hares,^32^ plants,^33,34^ forest species and forest type,^35^ macroinvertebrates,^36,37^ ungulates,^38^ songbirds,^39^ and a wide variety of other animals and fungi.^40,41^ Species range shifts in turn spur novel species interactions within native and expanded ranges^35,42,43^ and has the potential to alter or displace species’ functional roles within their ecosystems.^34,44,45^ For lakes specifically, warming temperatures influence community composition and biomass for diverse taxa.^46–48^ Additionally, changing lake stratification dynamics, and warming water temperatures coupled with increasing prevalence of lake browning is reducing availability of coldwater fish habitat.^49,50^

Novel conservation prioritization frameworks will assist practitioners in taking well-informed management action toward adapting to and mitigating increased velocity of change in accumulated heat on the landscape. More specifically, understanding how divergent ecosystems across mountain landscapes will respond to rising rates of heat accumulation anticipated by the end of the century will be important for deciphering which landscapes, and the terrestrial and aquatic species inhabiting them, may be most vulnerable to shifts.^51^ Managers, especially those tasked with conservation prioritization of sensitive systems, their flora, and their fauna, have relatively few tools or science-based strategies to triage their resources effectively.^52^ Therefore, a vulnerability classification of landscape regions based on heat accumulation and its velocity of change would be of wide appeal within the environmental management community.

In this study, we characterize climate vulnerability for all major USA mountain lake landscapes based on degree to which they have accumulated heat, historically and to end-of-century, as well as their experienced rate of change. Our specific goals were to (1) quantify heat, and harmful heat, accumulation across USA mountain landscapes over time. (2) Quantify experienced velocity of thermal change across these same landscapes. (3) Provide a mountain landscape classification based on heat accumulation such that any mountain landscape can be classified into one of three vulnerability types. (4) Quantitatively evaluate how landscape vulnerability, and classification, change over time under the modest SSP 3/RCP 7.0 climate scenario.

## Methods

### Data Sets

Spatialized lake polygon data for the United States (USA) were acquired from the National Hydrography Database (NHD) with the {nhdR} package (version 0.6.1).^53,54^ The NHD contains comprehensive and standardized spatial coordinate distributions of surface waters (e.g., lakes, ponds, streams, rivers, canals) throughout the USA. Only waterbodies with the “Lake/Pond” designation in the NHD were used in this analysis (0–497 km^2^ in surface area). The NHD was best suited for this study because it best captured mountain lakes, which are often small and miscounted, when compared to other popular databases.

Spatial NHD lake data (representing locations of “Watersheds” in the landscape, later joined to air temperature data) were joined to the Omernik Level III ecoregions framework (https://www.epa.gov/eco-research/ecoregions)^55,56^ and cropped to contain lake-watershed points within mountainous polygons for each of the 10 primary mountain ranges in the contiguous United States (Figure S1), contemporarily named: Appalachian/Atlantic Maritime Highland Mountains (*n* = 10,467), Arizona–New Mexico Mountains (*n* = 1033), Blue Mountains (*n* = 284), Blue Ridge (*n* = 464), Cascade Mountains (*n* = 2165), Idaho Batholith (*n* = 1035), Klamath Mountains (*n* = 245), Rocky/Colombia Mountains (*n* = 9661), Sierra Nevada Mountains (*n* = 2358), Wasatch–Uinta Mountains (*n* = 988). We note that these coordinates are meant to merely represent key locations on the landscape (i.e., “Watersheds” of the lake landscape) despite technically being linked to individual waterbodies for this analysis; thus sample size does not indicate total true total of lakes on those landscapes. Additionally, owing to restrictions of the NHD, surface area size cutoffs of the data, and the generally and notoriously poor ability to remotely sense small waterbody features in areas like mountains, this sample cannot represent an accurate count of lakes on the landscapes themselves. The ecoregions framework supports systematic ecological classification and aided spatially delineating USA mountain ranges. In instances where a lake boundary occurred in multiple ecoregions, and thus duplication occurred, the duplicate was removed. Lakes were assigned elevation data with {elevatr} (version 0.99.0).^57^

High resolution (30 arc sec, ∼1 km) global downscaled air temperature data were acquired from the open access CHELSA climate database (Climatologies at High resolution for the Earth’s Land Surface Areas; Version 2.1).^58–60^ Mean daily air temperatures (TAS air temperatures at 2 m from hourly ERA5 data) were acquired for both historical (1979–2019) and projected (2011–2040, 2041–2070, 2071–2100) time periods at the lowest provided resolution (monthly). The “business as usual” projected climatology (SSP 3/RCP 7.0) was selected for being the most realistic and policy-relevant scenario to achieve the goal of assessing heat accumulation in mountain landscapes. Historical data were available in unique year–month combinations (e.g., per lake, *n* = 456), but projected data, as is typical of climatologies, were available only as a conglomerative average across each time period–month for each unique SSP scenario (e.g., per lake, *n* = 12 for the 2011–2041 time period under SSP 3). The year 1979 was excluded from analyses due to incomplete data. Lake data from the NHD were joined to CHELSA data to acquire watershed-level air temperature values at the landscape level; this allowed for fine-scale assessment of landscape temperature change; however the method remains relatively limited in granularity (i.e., a large lake and adjacent pond are not comparable, and empirical measurements at these locations may show greater variability), thus we do not extend our interpretations to the site-specific scale for this analysis.

### Heat Accumulation

This study quantified growing degree days (GDD) and killing degree days (KDD) metrics for mountain landscapes in both historical and projected time periods (Figure 1; Figures S3 and S4). We calculated GDD for each unique Lake–Year–Month combination by adapting the standard degree days (DD) formula to

$$DD=\sum_{t=1}^{N}T_{t}-T_{0},T_{t}>T_{0}$$

where *N* = number of days, *T*_*t*_ = mean temperature on a day *t*, and *T*_0_ = threshold temperature beneath which thermal energy is considered negligible toward physiological growth and maturity processes of species in mountain landscapes, particularly aquatic species. To fit the structure of the data available for this study, we used the secondary equation and modified the following elements: *N* = number of months; *T*_*t*_ = mean temperature on a month *t*.

**Figure 1:**
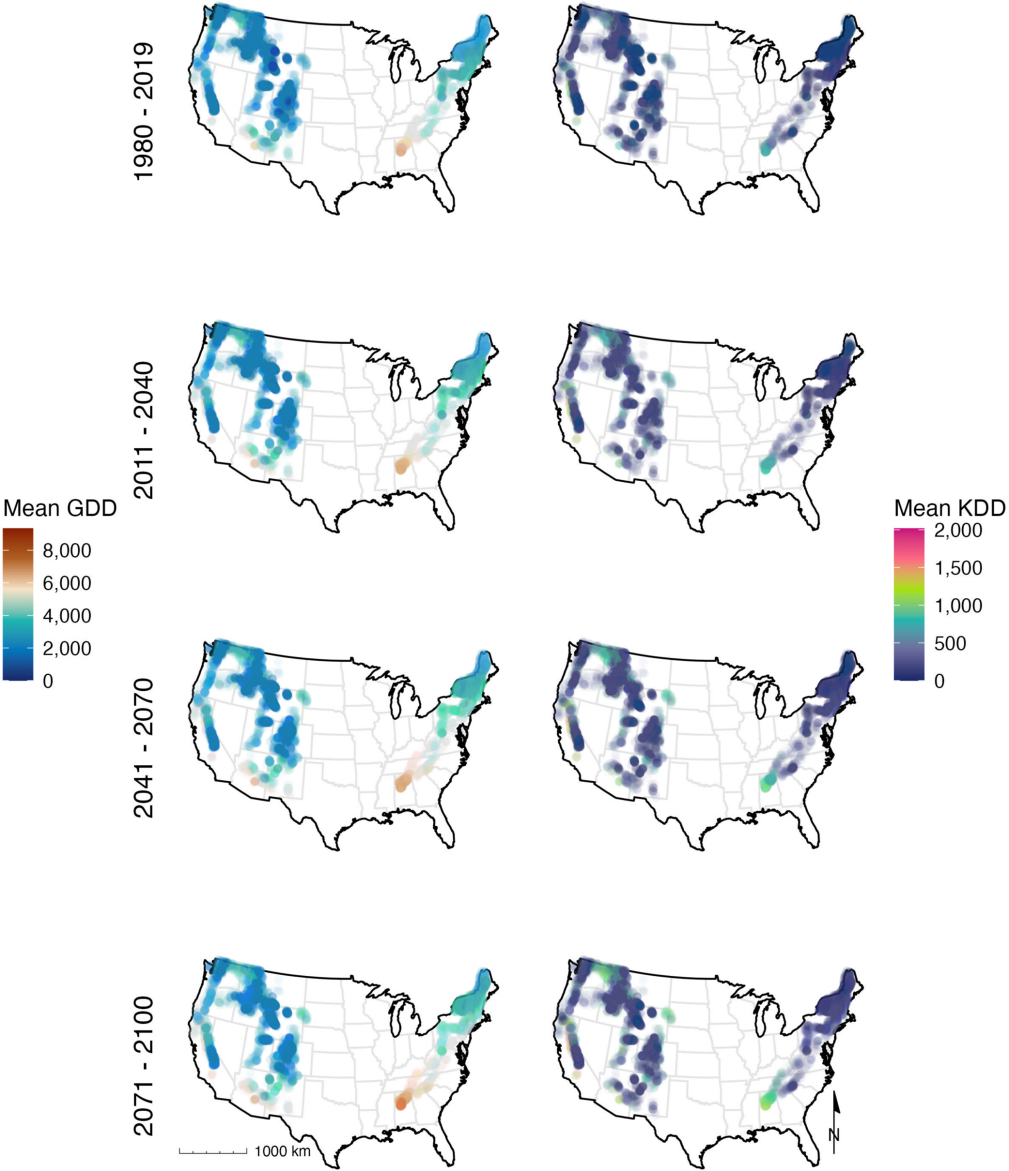
Mean growing degree days (GDD; left panel) and killing degree days (KDD; right panel) across mountain landscapes in the contiguous USA for the time periods: 1980–2019, 2011–2040, 2041–2070, 2071–2100 (top to bottom). Each unique map shows increasing GDD and KDD trends over time.

We applied a GDD threshold of 0 °C because it is the most parsimonious base temperature in general analyses of fish growth.^61^ We calculated KDD using the same equation but used the 90% quantile for each mountain range (13.25–22.85 °C) as the *T*_0_ threshold temperature; i.e., Appalachian/Atlantic Maritime Highland Mountains (20.4 °C), Arizona–New Mexico Mountains (20.9 °C), Blue Mountains (17.7 °C), Blue Ridge (22.9 °C), Cascade Mountains (14.7 °C), Idaho Batholith (13.2 °C), Klamath Mountains (18.7 °C), Rocky/Colombia Mountains (14.7 °C), Sierra Nevada Mountains (14.2 °C), Wasatch–Uinta Mountains (15.0 °C). While a range of definitions to define and calculate “unusual” or “extreme” heat exists across studies, the application of a 90% quantile approach is widely used.^62,63^ KDDs therefore broadly represent landscape temperatures that are, for native cold-adapted organisms at least, either lethal, near-lethal, or otherwise supraoptimal—conditions likely to impair organism growth, performance, or metabolic rates, though the precise effects are taxon and latitude dependent.^64,65^

When negative degree days resulted, these data were converted to zeros as it meant no heat had been accrued above the threshold. Observations where GDD = 0 or KDD = 0 were retained in the data set for modeling (see Methods “Linear mixed-effect models”). As CHELSA climate data were only available for unique Year–Month combinations, these data were expanded to complete the GDD or KDD calculation using mean monthly temperature as the expander for each month. Therefore, each day of a unique Lake–Year’s month received the same average temperature for each day of that respective month.^66,67^ Last, these expanded values were summed for every unique Lake–Year to acquire the number of growing or killing degree days for a watershed location in a year.

As elaborated upon in the discussion, lake surface water temperature (LSWT) generally corresponds with air temperature^68^ so as to understand general thermal trends occurring within natural landscapes. However, LSWT data are also not a substitute for lake temperature at depth, an ongoing challenge for lake landscape limnology. As this analysis focuses on landscape-scale patterns experienced in the microclimates of mountain watersheds, we do not suggest air temperature is any substitute for good empirical water temperature data collections. Furthermore, studies of actual patterns and trends in surface and hypolimnetic waters is essential to advancing this field in the future.^69,70^ Further, the approach used in this study, while focused on air temperature, could represent a “precursor approach” and thus eventually be applicable to empirical or remotely sensed water temperature data sets.

The GDD and KDD thermal metrics translate changes in temperature in the mountain landscape into ecologically meaningful interpretations. Both GDD and KDD are heat accumulation measures that have been broadly used for >70 years in ecology and >270 years in agronomy.^71–75^ While GDD and KDD are related, they have divergent ramifications for organisms. GDD measures cumulative heat units above a base threshold temperature, typically a threshold for growth and development.^61,75,76^ In contrast, KDD measures cumulative units over a known supraoptimal temperature threshold and is used to help assess cumulative risk of severe heat exposure to organisms. KDD is a related metric to those used in heatwave studies (e.g., ref 77) but emphasizes total accumulated heat as opposed to heat pulses. While GDD has long been applied as an ecological indicator in agricultural studies, it is generally underutilized in limnology and the aquatic sciences^74^ but see.^78–81^ Only relatively recently has the GDD concept been integrated into studies relating to zooplankton and phytoplankton,^82–84^ macrophytes,^85^ and freshwater bivalves.^86^

### Velocity of Climate Change

We measured velocity of change using {*lmerTest::lmer*} to run linear mixed effect models.^87^ We opted to apply this model structure because the goal of this study is not to compare the individual effects of air temperature in watersheds within a region (Zuur et al. 2009), but rather to estimate variation among watersheds. In the models, the estimates optimized the “restricted maximum likelihood” (REML) criterion, *GDD* for each Lake–Year combination was the response variable, Year was a fixed effect, and Lake was a random effect on both the slope and intercept (Table S2); we provide pseudo-*R* ^2^ values in place of random effect *p*-values, which is a recommended approach.^88–91^ The random effect slopes were subsequently interpreted as the velocity of change for each watershed. Overall trends in GDD were plotted with a black regression line and random effect slopes examined as a function of elevation for each mountain range (Figures 2 and 3). Using GDD slopes, we additionally show differences in the velocity of change for each mountain range; statistical significance of differences among mountain ranges was evaluated using one-way ANOVA (Figure 4). A parallel analysis was performed using annual mean temperature (°C) rather than GDD, and because similar trends resulted, we display only results from GDD for consistency with KDD analyses (Figures S5 and S6). Both model response variables were transformed, GDD ln­(*x* + 1) or temperature log­(*x* + 10), prior to modeling. The log­(*x* + 10) transformation was used to render all temperature values positive prior to taking the logarithm.

**Figure 2:**
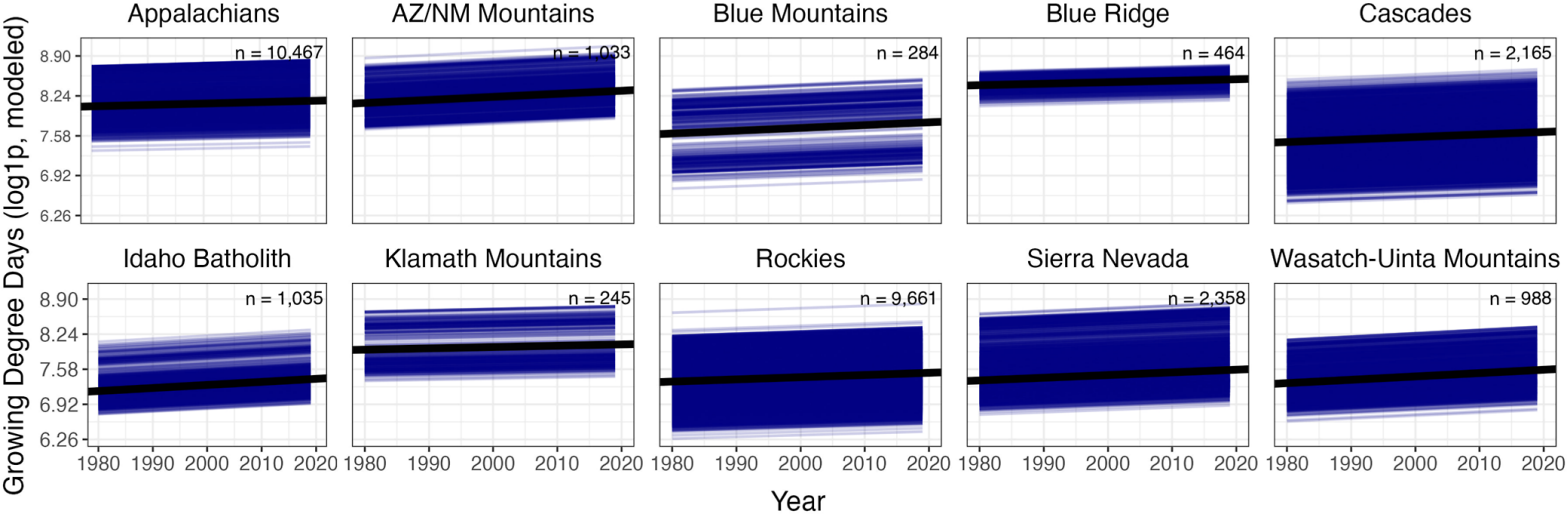
Long-term trends in GDD for mountain landscapes in 10 mountain ranges across the USA as assayed using random slope and random intercept linear mixed effect models. Black line denotes overall unique trend for each region. Thin blue lines represent distinct watersheds on the landscape. Sample size (*n*) denotes the number of points on the landscape used to construct the analysis.

**Figure 3:**
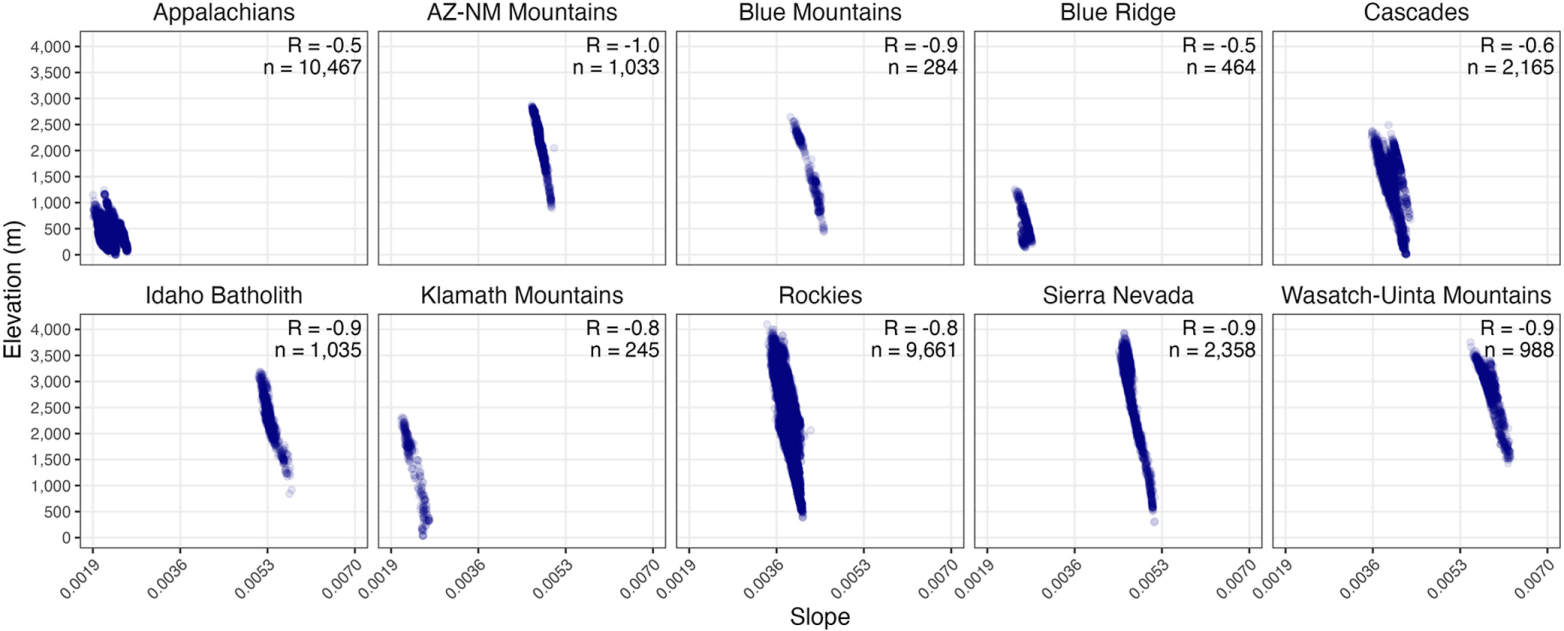
Velocity of climate change (assayed as random slopes extracted from the random slope and random intercept linear mixed effect modellog_10_ (GDD +1)) plotted against elevation of mountain lakes. Pearson correlation coefficient (*R*) is shown in upper right of each plot. Sample size (*n*) denotes the number of points on the landscape used to construct the analysis.

**Figure 4:**
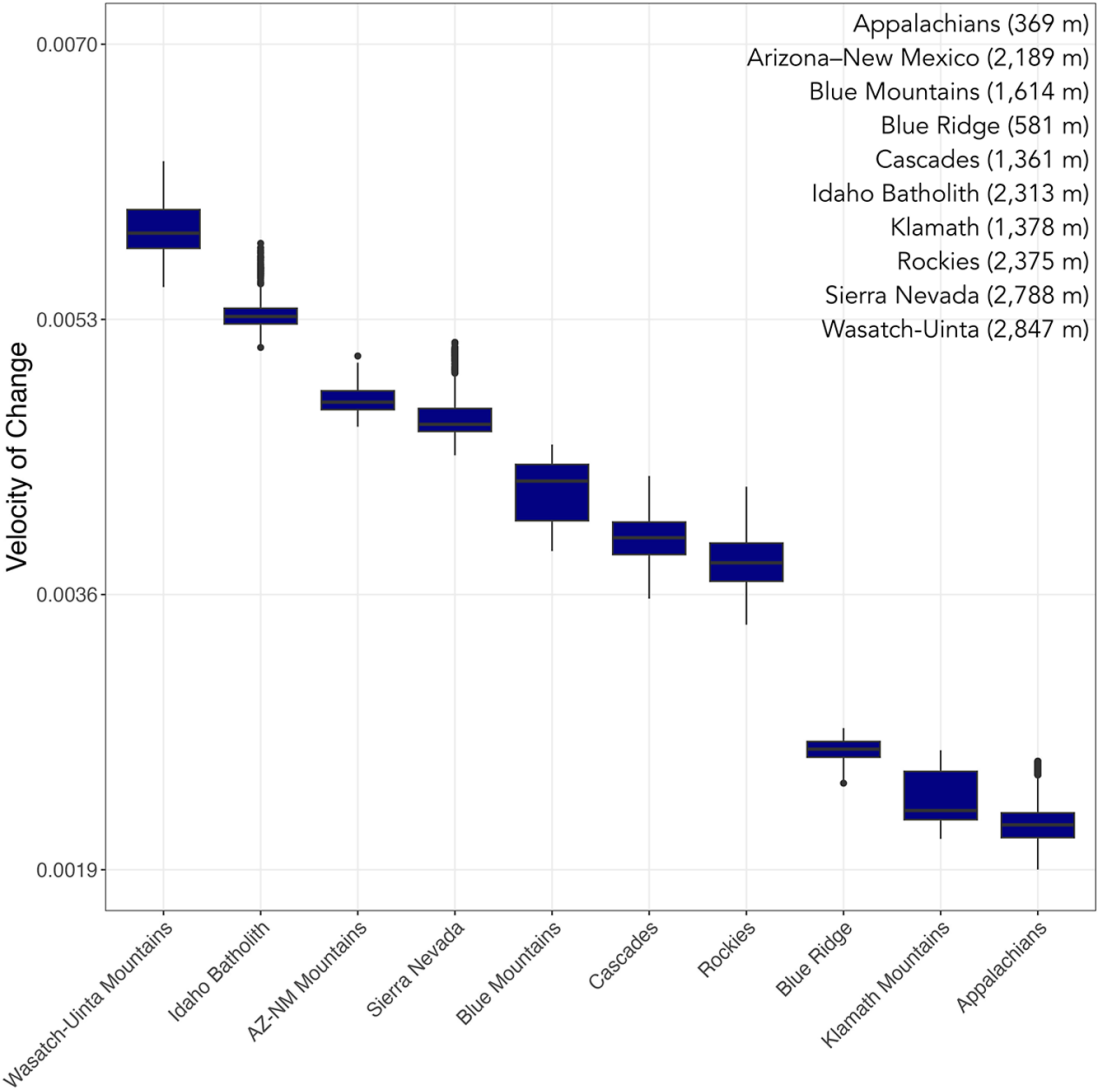
Box plots showing the range of observed velocities of change (random effect slopes, log_10_ (GDD +1)) in each focal mountain range. Each box represents the median value and interquartile range, and error bars denote the 95% confidence interval. Average elevation of the NHD locations within that range are featured in the upper right and Table S1.

### Climate Vulnerability Classification

We performed a k-means cluster analyses for each site based on hindcasted mean historical air temperature heat accumulation spanning the 38 y time series (1980–2019; mean GDD, ln­(*x* + 1) transformed). This was done to build the climate change vulnerability classification and to identify and group landscapes within each mountain range based on similar heat accumulation conditions experienced on the landscape. K-means is an ideal method for classifying rate of change in climate data as the method is versatile, guarantees model convergence, is scalable and computationally efficient with large data sets, and is simple and readily interpretable^92^ (Figure 5; Figure S7). The classification was a priori constrained to three clusters (i.e., cold, transitional, or hot). We elected not to cluster based on model slopes, primarily because the variance structure of projected climate data did not match that of the historical data sets. Hence given low sample size of projected data, and because GDD and slope are nearly colinear, we conservatively limited our analyses of slope to only historical data.

**Figure 5:**
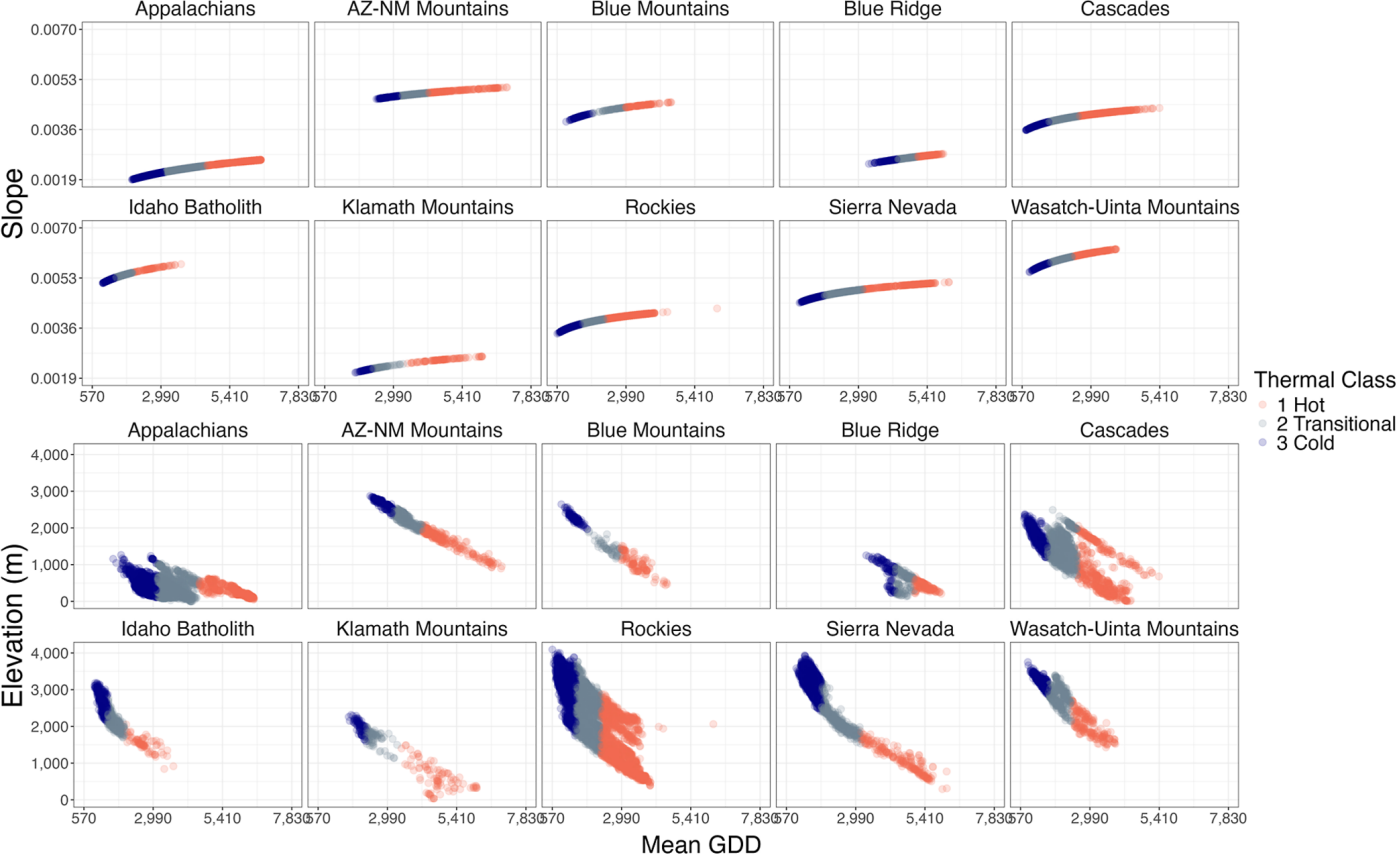
Relationship of velocity of change (random effect slopes, log_10_ (GDD +1)) and elevation as a function of mean GDD for each lake in 10 major mountain ranges in the USA. In each plot, each unique landscape is identified by its membership in each of the three climate vulnerability classes.

### Climate Change Projections

We performed discriminant function analyses (DFAs) to predict probability of lake assignment to one of the aforementioned clusters for three future time periods under the SSP 3 (RCP 7.0) climate scenario (Table 1; Table S3). DFA identifies the linear combinations of features that best separate the classes in the data set. DFAs were used to predict each ranges’ future cluster assignments and possessed a high degree of accuracy (>94%; Table S3). Each watershed’s mean historic GDD, and its respective cluster assignment, was used to build a predictive model for each mountain range separately. The continuous model variable, GDD, was ln­(*x* + 1) transformed as in the k-means cluster analysis, and scaled. Projected GDD for each mountain range was used to aid in cluster predictions. Analyses were performed using the linear DFA function from the {MASS} package (version 7.3-60.0.1).^93^ Using the above approach, we were able to successfully examine how climate vulnerability classifications changed given probable climate futures.

**Table 1:** Summary of Membership Totals (Historical and Future) in Each of Three Climate Vulnerability Classes for Major Mountain Ranges in the Contiguous United States, Including Percentage of Lakes (Non-Bold) and Percent Change (Bold) from the Historic Time Period

		percentage of lakes & percent change from historic baseline		
region	time period (1980–)	cold	transitional	hot
all	2019	42	39	19
	2040	25 (**−40**)	52 (**34**)	22 (**21**)
	2070	16 (**−62**)	57 (**46**)	26 (**43**)
	2100	8 (**−82**)	59 (**51**)	33 (**80**)
1. Appalachians	2019	45	44	10
	2040	26 **(−42)**	63 **(42)**	11 **(4)**
	2070	16 **(−66)**	72 **(62)**	12 **(21)**
	2100	5 **(−89)**	76 **(70)**	19 **(86)**
2. Arizona–New Mexico Mountains	2019	25	45	30
	2040	16 **(−36)**	47 **(4)**	37 **(24)**
	2070	7 **(−72)**	51 **(12)**	42 **(42)**
	2100	1 **(−98)**	45 **(−1)**	55 **(85)**
3. Blue Mountains	2019	40	19	41
	2040	38 **(−6)**	12 **(−34)**	50 **(21)**
	2070	36 **(−11)**	12 **(−34)**	52 **(26)**
	2100	23 **(−44)**	21 **(13)**	56 **(37)**
4. Blue Ridge	2019	23	44	33
	2040	8 **(−65)**	38 **(−14)**	54 **(64)**
	2070	4 **(−82)**	31 **(−31)**	65 **(99)**
	2100	1 **(−94)**	16 **(−64)**	83 **(153)**
5. Cascades	2019	28	48	24
	2040	17 **(−40)**	55 **(14)**	28 **(17)**
	2070	13 **(−55)**	56 **(15)**	32 **(35)**
	2100	7 **(−75)**	51 **(6)**	42 **(77)**
6. Idaho Batholith	2019	49	43	8
	2040	11 **(−78)**	80 **(84)**	10 **(29)**
	2070	4 **(−92)**	81 **(88)**	15 **(95)**
	2100	1 **(−99)**	75 **(74)**	24 **(221)**
7. Klamath Mountains	2019	30	33	38
	2040	9 **(−71)**	52 **(60)**	39 **(4)**
	2070	3 **(−90)**	57 **(75)**	40 **(7)**
	2100	1 **(−97)**	57 **(74)**	42 **(13)**
8. Rockies	2019	38	35	27
	2040	22 **(−41)**	44 **(26)**	33 **(25)**
	2070	15 **(−62)**	45 **(29)**	40 **(50)**
	2100	7 **(−81)**	47 **(32)**	46 **(73)**
9. Sierra Nevada	2019	67	24	9
	2040	54 **(−20)**	36 **(50)**	11 **(21)**
	2070	42 **(−38)**	47 **(96)**	12 **(32)**
	2100	26 **(−62)**	61 **(155)**	14 **(57)**
10. Wasatch–Uinta Mountains	2019	47	33	20
	2040	23 **(−51)**	50 **(52)**	27 **(37)**
	2070	14 **(−70)**	53 **(61)**	32 **(66)**
	2100	6 **(−87)**	55 **(68)**	38 **(96)**

### Data & Code Availability

Data materials used to construct this analysis are based on publicly available data cited in the manuscript text (i.e., NHD, Omernik, and CHELSA). Code to produce the main analysis, and Data Set S1, are available on GitHub (https://github.com/caparisek/mtn_landscape_heat_accumulation) and are registered on Zenodo (10.5281/zenodo.14954679). A conceptual figure illustrating the modeling steps is in the Supporting Information (Figure S8).

## Results

Statistical distributions in number and physical characteristics of individual lakes are quite variable across the study mountain ranges (Figure S2; Table S1). For instance, mountain ranges like the Appalachians and Rockies have numerically many more lakes compared with other ranges. These ranges, as well as the Sierra Nevada, also have more lakes with smaller surface area compared to larger ones, yet in contrast to these three ranges, ranges like the Appalachians have numerically many more low elevation lakes overall as the Appalachians are a relatively lower mountain range in general. Understanding the distribution of lakes across mountain ranges is primarily limited by the capacity of remote sensing tools to detect all small lakes.^94^ Nonetheless, with the data available, we observe lake surface area distributions of all mountain ranges are decidedly right-skewed, to varying degrees (Table S1). Trends in kurtosis (i.e., distribution tailedness) also shed light on how rare large lake ecosystems (e.g., Lake Tahoe, 496.2 km^2^) are across mountain ranges. While all ranges exhibit leptokurtic distributions (i.e., kurtosis >3, sharp peak in small lakes with long, thin tails toward larger lakes), the degree to which they exhibit this varies greatly.

### Heat Accumulation

In all mountain ranges, mean growing degree days (GDD) and killing degree days (KDD) increased over the historical period (1980–2019), and from the historical baseline to 2100 in the projected SSP 3 (RCP 7.0) climate scenario (Figure 1). Based on downscaled historical climate data, lakes in low elevation watersheds are consistently exposed to a greater number of GDDs than high elevation lakes; this pattern was present in all mountain ranges (Figure S3). Methodologically, the KDD threshold was unique for each mountain range, and interestingly, a range of midhigh elevation sites experience low KDD with sites at lower elevations often having the highest KDDs. In some cases, there was a tight relationship between elevation and KDD (e.g., Sierra Nevada, Blue Ridge), but in others, the relationship was more variable than this (e.g., Cascades, Rockies). Similar heat accumulation trends and an increase in KDD over time are also evident in the future (Figure S4). Quantiles derived from historical climate data illustrate the distributions of air temperatures within these diverse watershed landscapes (median = 4.75 °C, interquartile range = −3.15 to 12.95 °C).

### Velocity of Climate Change

Mixed-effect models examining relationships between historical year and GDD (heat accumulation) revealed increasing trends in every mountain range (*R*_c_^2^ > 0.89 (i.e., *R* ^2^_c_ is the variance explained by both fixed and random effects relative to total variance); Table S2; Figure 2). This pattern was almost identical for models constructed using annual mean temperature (°C) in place of GDD (Figure S5). Slopes extracted from these models for each site (as random effects), allowed comparisons of velocity of change estimates across sites (Data Set S1). For both GDD and temperature models, and across all mountain ranges, velocities of change correlated significantly with elevation (Figure 3; Figure S6; Pearson correlations: all correlations −0.95 to −0.48, all *p*-values <0.0001). Thus, watersheds with the highest velocity of climate warming tended to be those distributed at lower elevations.

Boxplots examining GDD-modeled slope as a function of mountain range indicate which landscapes experience faster rates of change compared to others (one-way ANOVA: *F*(928,690) = 193,288, *p* < 0.001). For example, the Wasatch-Uinta, Idaho Batholith, Arizona-New Mexico, and Sierra Nevada Ranges are changing most quickly, while the Blue Ridge, Klamath, and Appalachian Ranges appear to be changing relatively more slowly (Figure 4).

### Climate Vulnerability Classification

We built a climate change vulnerability classification using hindcasted air temperature heat accumulation data spanning a 38 y time series. Thus, every modeled mountain landscape was identified and subsequently its watershed sites clustered into one of three classes of climate vulnerability: (1) cold, (2) transitional, or (3) hot (Figure 5; Figure S7). Across all mountain ranges 1980–2019, 19% of sites held characteristics consistent with high heat and fast rates of heat accumulation, 42% of sites remain colder with slow rates of change, and 39% of sites are classified as transitional (Table 1). The percentage of watersheds assigned to each of these categories varied for each mountain range, such that historically the Sierra Nevada had 68% of its watersheds classified as cold, and Idaho Batholith, Wasatch-Uinta, and the Appalachians had 48%, 47%, and 45%, respectively. In contrast, ranges such as Blue Ridge and Arizona-New Mexico had 22–25% of watersheds classified as cold. However, these proportions change dramatically over time with probable climate projections (see Climate Change Projections below).

### Climate Change Projections

DFAs for each mountain range performed exceptionally well (>94% accuracy, *p* < 0.0001; Table S3). DFAs revealed that by the end of the century just 8% of sites across all ranges will be classified as cold, 33% of sites will likely be classified as hot, and 59% of sites will be transitional (Table 1). This represents changes of −82%, +80%, and +51%, respectively, from the historical baseline. Ranges such as Blue Ridge, Idaho Batholith, and Klamath, are anticipated to have just 1% of “cold” landscapes left by the end of the century, with the Appalachians, Cascades, Rockies, and Wasatch–Uinta having just 8%, 7%, 7%, and 6% of cold landscapes remaining (Figure 4).

## Discussion

Landscape differences in geology, latitude, and longitude promote differences in the ecology of lakes.^95,96^ In this study we (i) quantified heat accumulation and velocity of change across mountain landscapes in the USA and found that lower elevation landscapes, and those with greatest historical velocities of change, are most vulnerable to high heat accumulation.^97,98^ Further, the percent of mountain watersheds classified as highly vulnerable is anticipated to increase from 19% to 33% by the year 2100. Additionally, we (ii) investigated the potential of applying the agro-climate thermal time indicator, killing degree days, specifically to the landscapes of lake watersheds, and found that the percent change in mean killing degree days will increase, on average, by 236% by the year 2100. We also (iii) created a climate change vulnerability framework to assist decision makers in the allocation of their limited conservation resources toward these sensitive environments.

Thermal extremes in freshwaters are increasing in frequency and threaten aquatic organisms and ecological processes as end-of-century approaches.^99–101^ In high-altitude ecosystems, snowpack is diminishing and ice-cover on lakes is reducing rapidly; this alters water security downstream and wreak havoc on thermal regimes in these coldwater habitats.^50,102–104^ Higher heat accumulation in lakes is also known to increase disease susceptibility,^105^ favor phytoplankton blooms,^106^ modify lake stratification dynamics,^107^ and reduce oxygen levels in lakes,^108,109^ all of which could disrupt or rewire food webs.^110^ Populations of a species that experience different levels of temperature variation across a landscape will likely develop different thermal tolerances and have altered thermal ranges over time.^111–113^ Some taxa, like some lake-dwelling mountain aquatic insects, may be able to mitigate risk of heat exposure in lakes by migrating to cooler refugia (e.g., spring- or snowpack-fed streams) if required.^114,115^ Additionally some terrestrial insects and arthropods may have the ability to disperse as well.^15^ However, other taxa may be unable to effectively disperse to more favorable habitats, especially if lakes are not hydrologically connected, and so both dispersal ability and the landscape-specific context of lakes will be important in determining ultimate changes in diversity.

In this study, we predict mountain landscapes that previously supported more favorable coldwater habitats will experience more days with higher temperatures, greater accumulated heat, and an amplification of killing heat. Where landscapes newly experience greater growing degree days, these warmer temperatures may open up novel habitats suitable to support optimal growth and development in the future. However, we also predict these landscapes will experience 215–254% (mean = 236%) increases in heat accumulation exceeding the 90th percentile historical temperatures. Our findings suggest that across USA mountain ranges, watersheds positioned at lower elevations are consistently exposed to higher rates of heat accumulation. This latter point, despite being based on air temperature data, is also supported by observed trends in surface water temperature from some mountain ranges, such as the Pyrenees.^116^ The accumulated heat (i.e., degree-day) metric is a valuable tool for assessing changing heat content dynamics.^117,118^ In freshwater systems generally, increased heat accumulation extends the duration of the growing season and can enhance maturation rate in fishes;^78,119^ however, some fish populations have lower tolerance to high temperatures and, consequently, perform less well.^120,121^ Indeed, research suggests ecological response to increased heat accumulation is nonlinear, as it is also known to be ecosystem-specific and heavily associated with changes in latitude.^80,122–124^ It is unknown how fishes and other aquatic organisms respond to heat accumulation along an elevation gradient. For instance, organisms may attempt to migrate or else attempt to tolerate warming temperatures. Relatedly, climate change may simultaneously increase primary productivity and thereby improve food resources for higher order taxa in the food web.

Quantifying geographically distinct velocities of climate change provides critical insight and nuance on the uneven impacts of climate change. For example, we observe that velocity of climate change varies considerably by mountain range (i.e., some ranges experience greater velocities of change through time, while others have relatively slower rates of heat accumulation). This finding provides key insight on the fragility of certain regions and lakes to ecosystem state shifts.^25,27,125^ Individual species and ecosystems possess different thresholds for how they will react to higher heat accumulation; however, the pace at which they can acclimatize to the rapidity of these changes is also important. Species with less time to adjust to rapidly increasing temperatures (e.g., long-lived and less mobile organisms), are likely to struggle in climates whose heat accumulation occurs at a higher velocity.^126,127^ However, a slow rate of change can also be dangerous, especially in regions where climate variance has historically been low.^128^ Likewise, populations of a species experiencing thermal variability will have differing thermal ranges.^111–113^

While GDD and velocity of change are closely linked, the relationships are apparently often curvilinear (e.g., Appalachians, Cascades, Sierra Nevada; Figure 5). Therefore, velocity of change actually slows once a threshold of high heat accumulation is reached. This pattern is consistent with expectations from regime shift theory, where the highest rates of change are more frequently observed in systems undergoing a state shift.^125^ Combined, the empirical patterns in velocity of thermal change suggests these landscapes have likely been rapidly shifting for some time, so much so perhaps, that the rate of change is actually beginning to slow. These relationships importantly highlight how heat accumulation and velocity of change are fundamentally different assessments of vulnerability that can sometimes, though not always, be correlated with one another.^129–131^ Some of our study mountain ranges showed parallel results in their heat accumulation and velocity of climate change (e.g., Wasatch–Uinta Mountains) while in others, heat accumulation and velocity of climate change were decoupled (e.g., Klamath Mountains). Therefore, conservation applications based on just one or the other may come to divergent conclusions. Coupling velocity of change with heat accumulation provides a richer portrait of vulnerability, which may be of interest in future climate change assessments efforts going forward.

A limitation of our analysis is the lack of available water temperature data, a problem that is exacerbated by the lack of study in mountain systems more generally. These data are not yet feasible to acquire at scale, and so here we used air temperature data to explore changing patterns in accumulated heat in the landscape. There is evidence that lake surface water temperature (LSWT) does generally correspond closely with air temperatures^68^ and thus can still be a useful proxy, specifically for nontaxa-specific landscape-level temperature-based analyses. While LSWT cannot serve as a proxy for lake temperature at depth, and attaining lake depth temperature estimates at scale remains elusive to scientists, this information still provides valuable insights into microclimates experienced in mountain watersheds. Future work could build from this study by forging models on well-studied lakes that generate hindcasted and forecasted lake temperatures^69,70^ rather than just landscapes.

It is worth noting that lakes themselves do not necessarily show the same temperature trends as their watersheds, and thus these results should only be interpreted as landscape-level trends. As demonstrated by Figure S2 and Table S1, while most mountain ranges are indeed skewed toward having smaller waterbodies, outlier lakes that are very large are also present (e.g., Lake Tahoe, in the Sierra Nevada mountains). Factors contributing to the lake heat budget, such as duration of ice cover, the water color and the resulting attenuation coefficient of radiation, lake morphology such as surface area and volume, and exposure to solar radiation, cloud cover, and albedo effects, play key roles in making lake warming not a geographically consistent phenomena (O’Reilly et al. 2015). Additionally, while high elevation mountain lakes may experience greater elevation-dependent warming throughout the day, reduced snow cover in a given year coupled with greater solar radiation will drive convective cooling (i.e., night time heat loss) which plays a large role in the actual water temperatures in mountain lakes. Seasonal effects, such as the ice-free season leading to more warming in the summer and ice and snow cover enhancing colder temperatures in the winter, also play significant roles in mountain lake temperatures.^132^ Thus, even though mountain lakes should be experiencing high rates of elevation dependent warming, factors such as the timing and volume of snowmelt, the duration of the ice-free season, and the magnitude of convective nighttime cooling all can play important roles in lake heat budgets, causing lakes to warm at rates slower than would be expected. Finally, we note the relationship between lake surface area and elevation is quite varied across the ranges (Figure S2, panel D). This variation would likely present differences in lake heat budgets as well. This area of research would benefit from having the ability to tease apart nuances such as lake volume, maximum depth, morphology, and convective cooling, as these could all reasonably influence the speed at which lakes accumulate heat as landscape temperatures rise.^116^

Ecosystem vulnerability assessments are core to advancing conservation activities at many scales.^133–136^ The goal of our proposed climate change classification is to help identify, across multiple mountain ranges, the vulnerability of individual mountain landscapes to increasing heat accumulation. The three clustering tiers are delineated by (1) low heat accumulation, often with sites from high-elevation; (2) transitional, often with sites from midelevation; and (3) high heat accumulation, often with sites from lower elevation ranges. Combined, the classification schema shows lower-elevation mountain lakes are experiencing more rapid landscape-level thermal change across all USA mountain ranges. These lakes are also most likely to first experience increased killing degree days as end of the century approaches. Further, our findings suggest particular conservation consideration should be given to watersheds that have fewer than 5% of cool landscape available to them by the end of the century (e.g., Appalachians, Blue Ridge, Idaho Batholith, Klamath) because this restricts the occurrence of cold-adapted endemic species.

Accelerating change in freshwater systems will force managers to strategically select where they can reasonably work for maximal impact. The vulnerability schema provided here provides an initial tool to help. Global lake thermal regimes are already undergoing worldwide shifts at increasing velocities.^130,137^ No study exists, however, which classifies landscape vulnerability in mountain regions for anticipated heat accumulation and rates of change. Previous accepted frameworks for lake thermal classification exist, although they emphasize mixing regimes and require specific data to perform multidimensional lake models.^138–140^ To assess landscape vulnerability at scale, however, these data are not available and thus application of these frameworks are limited. Numerous assessments have sought to quantify vulnerability of lakes, depending on the focal need of the assessment, including through change in eutrophication,^136^ pollution resilience,^141^ water balance,^142^ and invertebrate-based temperature reconstructions.^143^ Some studies have concluded high-elevation lakes to be most vulnerable to change when specifically focusing on changes in ice dynamics, which low elevation lakes do not frequently experience.^144,145^ However, assessments using the accumulated degree-day approach supports our finding that low-elevation watersheds are indeed highly sensitive to warming trends.^116,146^

There are several potential uses for our mountain landscape thermal classification framework. Many of the most well-studied mountain lakes are located at relatively high elevations in their mountain ranges. Results from this study suggest managers should increasingly monitor coldwater lakes at lower-to-mid elevations. Further, while shallow versus deep lakes would be affected on the landscape differently, these watershed locations still are mostly likely to experience the greatest accumulated landscape heat. Regional managers can use our classification to identify specific watersheds of greatest threat to loss of endemic species. Further, the classification provides an initial ability to better understand types of challenges these species are uniquely facing (e.g., fast change or slow change) and thus provides an ability for managers to take early action in watersheds undergoing the greatest threats to species. Yet whereas climate change itself is unmanageable at a local scale, conservation practitioners must find ways of building resilience into ecosystems using the levers that they do have control over.^147^ For some watersheds, this might mean reduced harvest limits or improved in-lake or shoreline habitats.^28^ In other ecosystems, it may entail improved management of the watershed, land use and nutrient loading.^50,148,149^ We therefore encourage managers to use the information provided here to plan resource allocation, funding needs, and decision making toward climate change resilience.

Freshwater biodiversity is increasingly challenged by the scope and extent of global climate change and human domination of the world’s water cycle.^1–3^ This analysis provides an initial attempt and novel perspective to understand landscape vulnerability across USA mountain ranges. Our results show how vulnerable mountain lakes are experiencing unprecedented exposures to heat accumulation, especially at low elevations. Increased velocities of change are also fundamentally reshaping the structure and function of these ecosystems and increasing their frailty. Conservation managers need tools to prioritize their time, energy, personnel, and budget. In providing this classification and vulnerability analysis of the USA mountain landscapes, we hope to deliver one useful tool for aiding in complicated decision-making processes. Overall, our results call attention to the wide ways in where mountain landscapes are likely to change in the next 75 years.

## Supplementary Material





## Data Availability

This work is based on publicly available data cited in the manuscript text. Code used to produce the main analysis, and Data Set S1, are available on GitHub and registered on Zenodo (https://github.com/caparisek/mtn_landscape_heat_accumulation and 10.5281/zenodo.14954679, respectively). EarthArXiv, 10.31223/X5V429.

## Notes

Data set S1 (XLSX)
Figures and Tables (PDF)
