# Supplementary material for: Velocity of Climate Change and the Vulnerability of Mountain Lake Landscapes
Source: Environ Sci Technol. 2025 Aug 15;59(33):17507–20. doi: 10.1021/acs.est.5c03154 (PMC12404182; doi:10.1021/acs.est.5c03154)
Supplement: Supplementary file 2 [file es5c03154_si_002.pdf]

Supporting Information

Velocity of climate change and the vulnerability of mountain lake landscapes

Christine A. Parisek[1,2,*], Jonathan A. Walter[2,3], Steve Sadro[4], Andrew L. Rypel[1,2]

[1] University of California Davis, Department of Wildlife, Fish, & Conservation Biology, Davis, California, 95616, USA

[2] University of California Davis, Center for Watershed Sciences, Davis, California, 95616, USA

[3] University of Virginia, Department of Environmental Sciences, Charlottesville, Virginia, 22903, USA

[4] University of California Davis, Department of Environmental Science & Policy, Davis, California, 95616, USA

* Corresponding author

Author emails & ORCID
CAP: caparisek@ucdavis.edu / https://orcid.org/0000-0002-7648-879X
JAW: jawalter@ucdavis.edu / https://orcid.org/0000-0003-2983-751X
SS: ssadro@ucdavis.edu / https://orcid.org/0000-0002-6416-3840
ALR: rypel@ucdavis.edu / https://orcid.org/0000-0002-9812-8306

Running head: Mountain landscape heat accumulation

Keywords: Freshwater, mountain landscapes, high elevation lakes, climate change vulnerability, heat accumulation, velocity of climate change, speed of thermal change, growing degree days, killing degree days

SI document summary:

13 pages total

1 page Summary of Supporting Information

8 figures (Supporting Figures S1 – S8)

3 tables (Supporting Tables S1 – S3)

1 dataset (Dataset S1)

Summary of Supporting Information

Supporting Figure S1: Map of 10 focal mountain ranges used in this analysis.

Supporting Figure S2: Frequency histograms for temperature, lake surface area, and elevation, for lakes in 10 USA mountain ranges.

Supporting Figure S3: Historical sum of growing and killing degree days as a function of elevation.

Supporting Figure S4: Projected killing degree days as a function of elevation.

Supporting Figure S5: Temporal trends in temperature for mountain landscapes in 10 USA mountain ranges.

Supporting Figure S6: Velocity of temperature change (via slopes) plotted against elevation.

Supporting Figure S7: Boxplots of mean GDD, mean KDD, elevation and veleocity of change in each lake landscape and based on climate vulnerability category.

Supporting Figure S8: Conceptual figure of the methodological steps of the analysis.

Supporting Table S1: Descriptive statistics of statistical moments for surface area and elevation of lakes in each mountain range.

Supporting Table S2: Summary statistics for linear mixed effect regression models.

Supporting Table S3: Summary statistics for discriminant function analyses.

Supporting Dataset S1: Data materials combining NHD, Omernik, and CHELSA, including slopes, intercepts, and descriptive information for each NHD lake watershed.

Supporting Figures & Tables

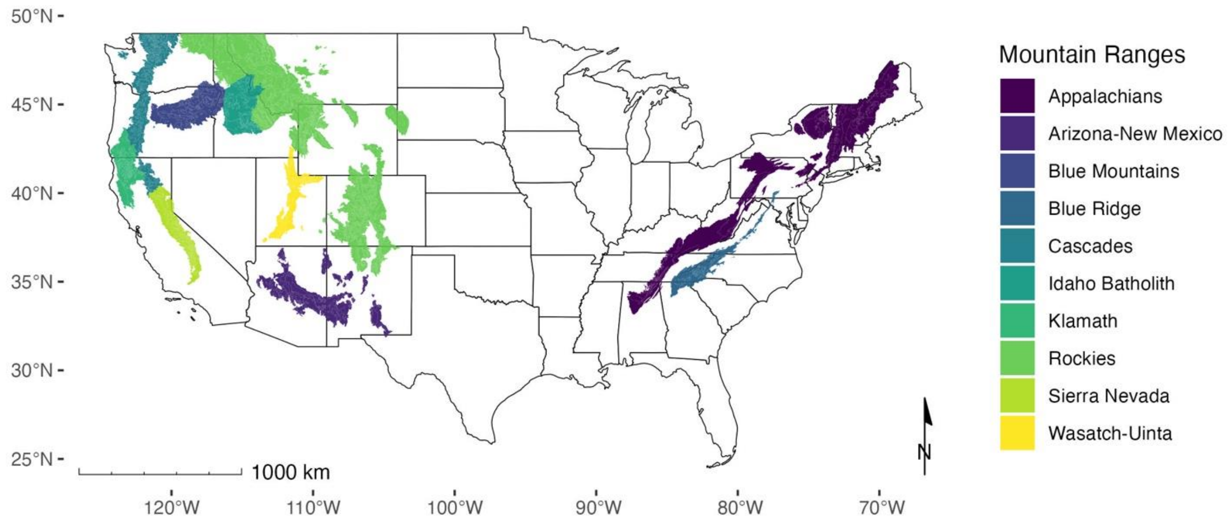

Figure S1

Map of the contiguous United States of America showing the ten focal mountain ranges in this study.

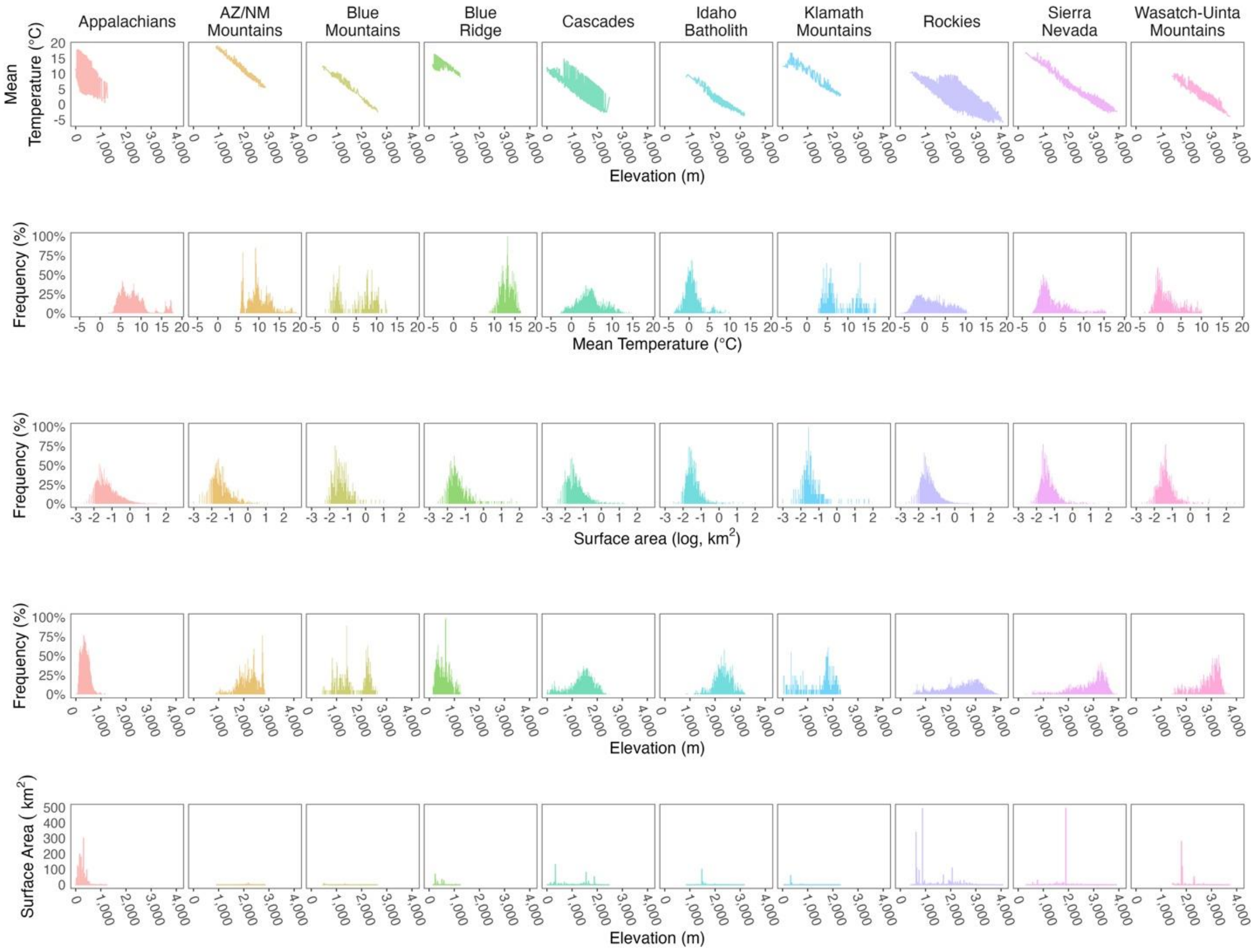

Figure S2

Frequency histograms for temperature (°C), lake surface area (log, km^2), and elevation (m) for lakes in 10 USA mountain ranges. Surface area as a function of elevation is shown to provide context and highlight lake diversity across ranges. Data were obtained from the National Hydrography Database (NHD) and CHELSA Database. Plotted temperature represents the average temperature 1980–2019 for a lake point in the NHD.

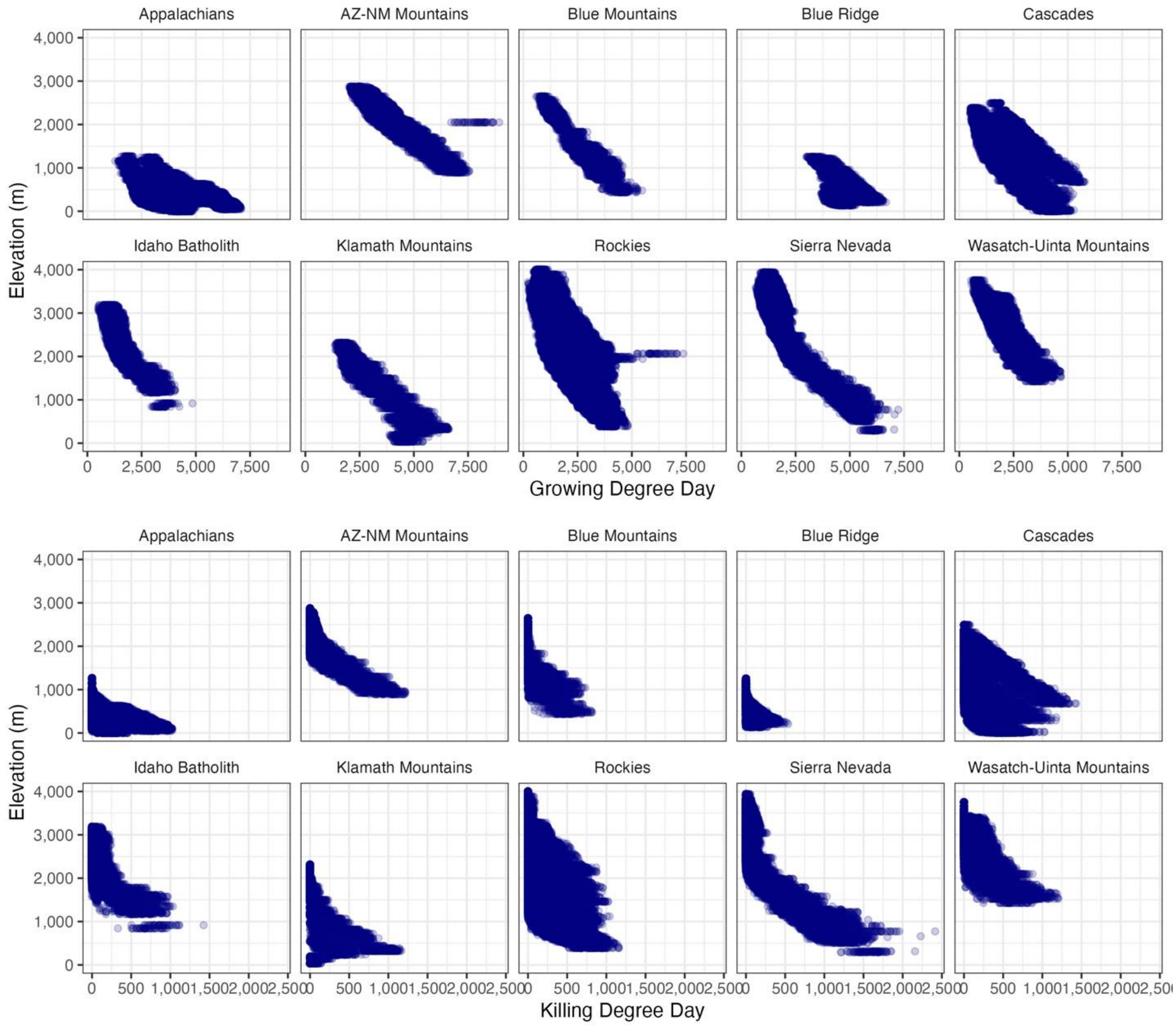

Figure S3

Historical sum of growing degree day and killing degree day as a function of elevation. Each point represents a unique *Lake–Year* combination.

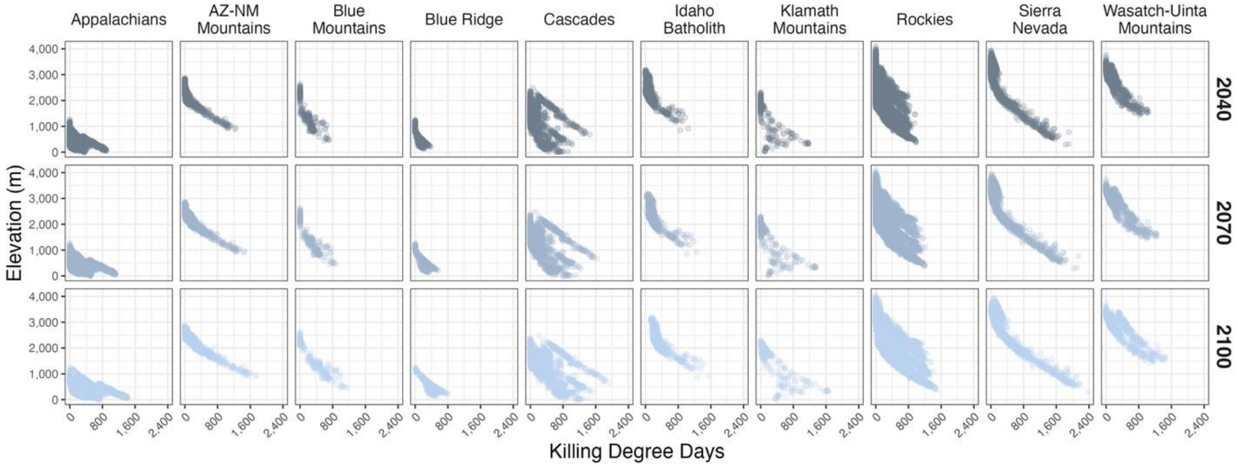

Figure S4

Projected killing degree days as a function of elevation. Each point represents a unique *Lake–Year* combination.

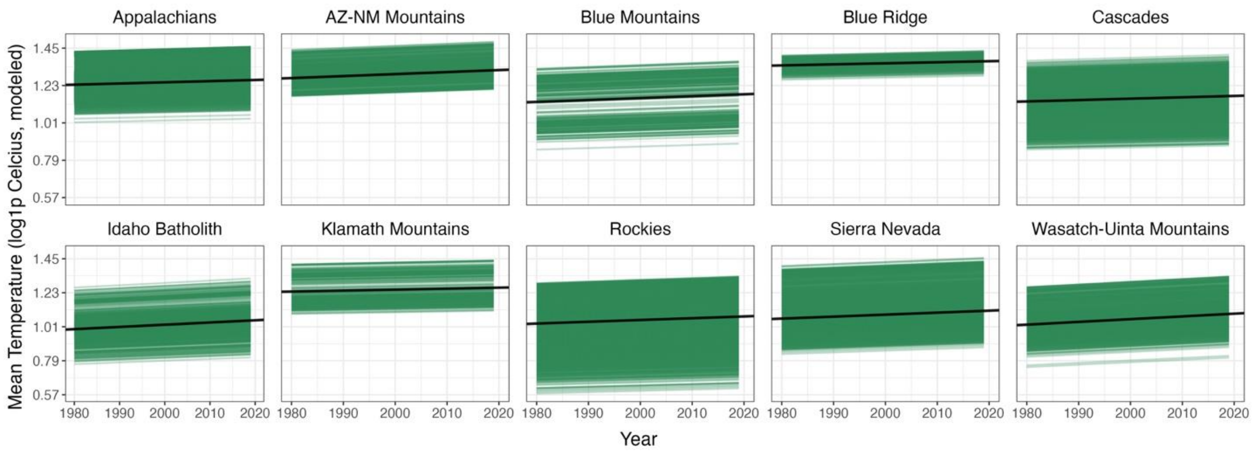

Figure S5

Long-term trends in temperature for mountain landscapes in 10 mountain ranges across the USA as assayed using random slope and random intercept linear mixed effect models. Black line denotes overall unique trend for each region. Thin green lines represent distinct watersheds on the landscape.

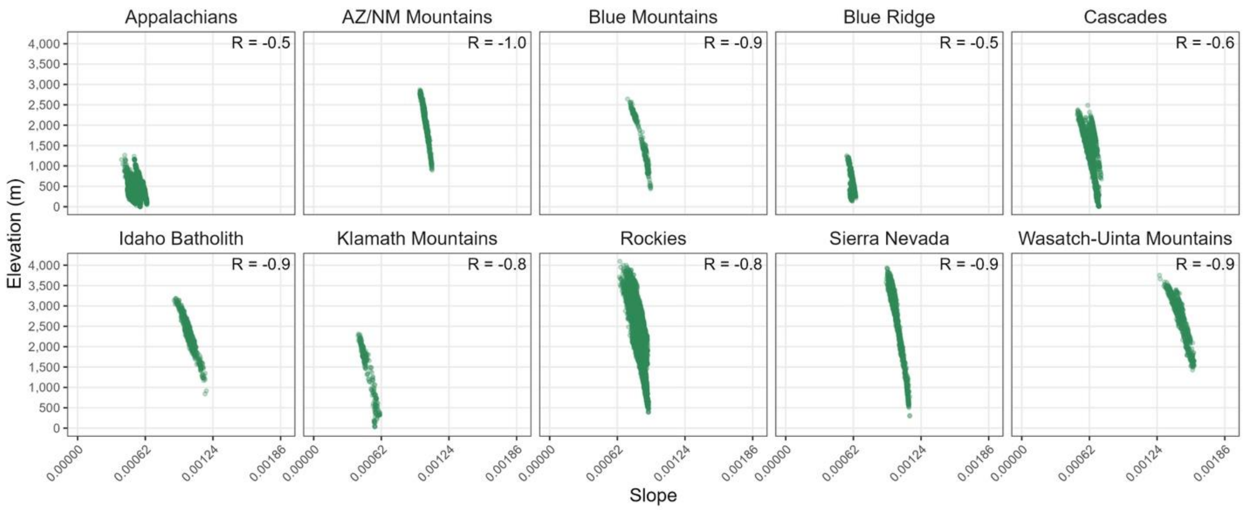

Figure S6

Velocity of temperature change (assayed as random slopes extracted from the random slope and random intercept linear mixed effect model, $\log_{10}$(Temperature ^{o}C+10) plotted against elevation of mountain lakes. Pearson correlation coefficient (R) is shown in upper right of each plot.

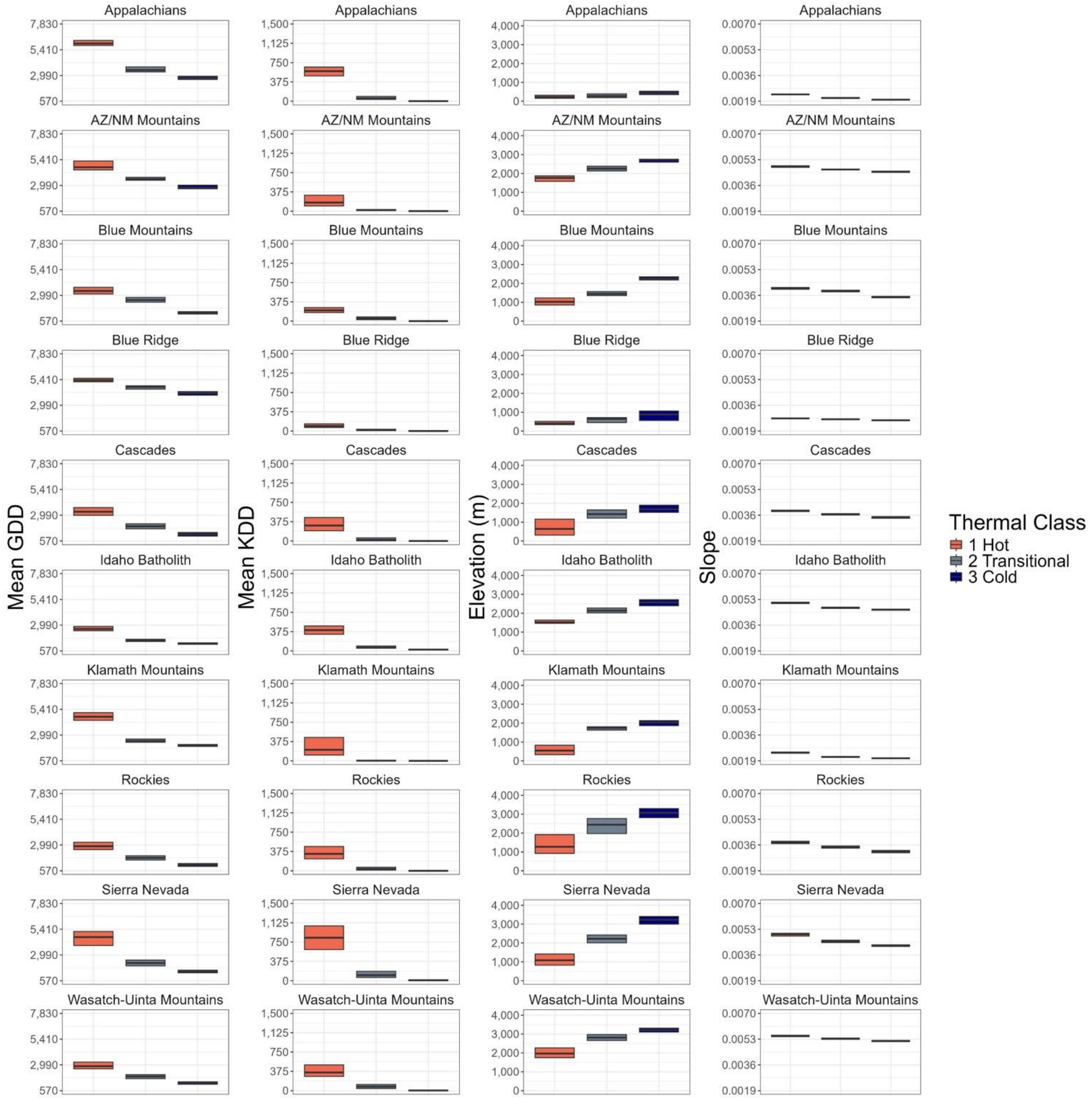

Figure S7

Boxplots of mean GDD, mean KDD, elevation, and velocity of change (random effect slopes, $\log_{10}$(GDD+1)) in each landscape and climate vulnerability category. In each plot, boxes represent the median and interquartile range.

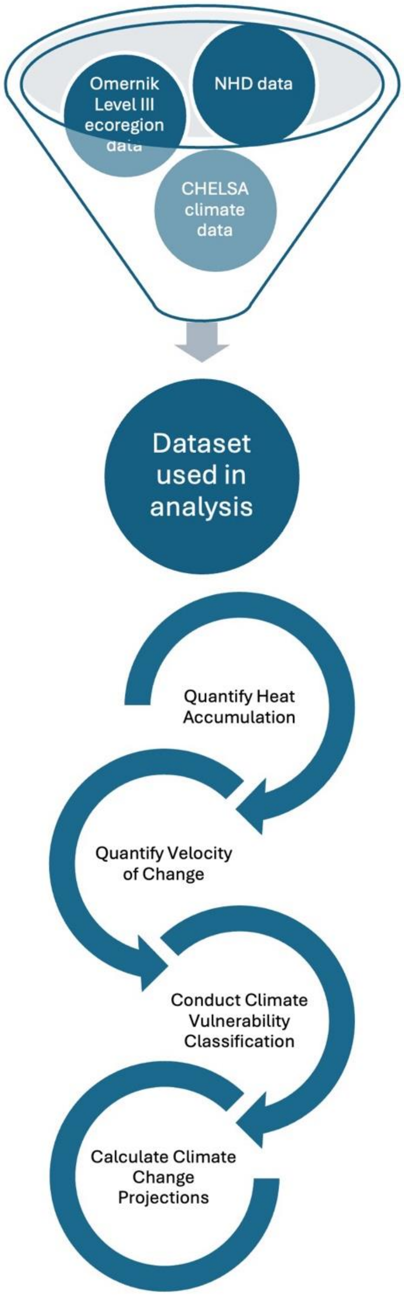

Figure S8

A conceptual figure illustrating the three datasets used and the order in which they were integrated into the modeling steps for their analysis products to generate the final climate change projections.

Table S1

Descriptive statistics of statistical moments for the distributions of surface area (km^2) and elevation (m) for each studied mountain range.

Mountain Range	Lake Abundance (n)	Surface Area (km^2)			Elevation (m)		
		Skew	Kurtosis	Mean	Skew	Kurtosis	Mean
1. Appalachians	10,467	33.3	1,376.3	0.4	0.7	4.0	369
2. Arizona–New Mexico Mountains	1,033	17.9	418.3	0.1	-0.6	3.1	2,189
3. Blue Mountains	284	10.9	134.3	0.1	0.0	1.6	1,614
4. Blue Ridge	464	11.2	150.1	0.6	0.5	2.5	581
5. Cascades	2,165	26.9	844.4	0.3	-0.7	3.0	1,361
6. Idaho Batholith	1,035	29.4	908.9	0.2	-0.4	3.5	2,313
7. Klamath Mountains	245	10.7	128.9	0.6	-0.6	1.9	1,378
8. Rockies	9,661	62.1	4282.3	0.3	-0.5	2.3	2,375
9. Sierra Nevada	2,358	47.8	2305.4	0.4	-1.1	3.8	2,788
10. Wasatch-Uinta	988	25.1	681.2	0.6	-1.0	3.1	2,847

Table S2

Summary statistics for linear mixed-effect regression models (*Temperature ~ Year (Year | LakeID)*) from which velocity of climate change metrics were extracted.

Mountain Range	Pseudo R^2	Parent Intercept	Parent Slope	Model Slope (Year Effect) df	t-value	p-value
	**Growing Degree Days (1980–2019)**					
1. Appalachians	0.97	3.48	0.0023	1,142	370	<0.0001
2. Arizona–New Mexico Mountain	0.96	-1.86	0.0050	37,799	226	<0.0001
3. Blue Mountains	0.97	-1.53	0.0046	2,120	70	<0.0001
4. Blue Ridge	0.89	3.56	0.0025	8,434	93	<0.0001
5. Cascades	0.95	-0.79	0.0042	10,022	150	<0.0001
6. Idaho Batholith	0.86	-4.21	0.0057	39,612	132	<0.0001
7. Klamath Mountains	0.97	3.07	0.0025	1,858	39	<0.0001
8. Rockies	0.95	-0.82	0.0041	371,846	297	<0.0001
9. Sierra Nevada	0.96	-2.62	0.0050	6,879	201	<0.0001
10. Wasatch-Uinta Mountains	0.94	-5.14	0.0063	18,566	159	<0.0001
	**Temperature (1980–2019)**					
1. Appalachians	0.96	-0.14	0.0007	135,980	313	<0.0001
2. Arizona–New Mexico Mountain	0.96	-1.06	0.0012	94,82	188	<0.0001
3. Blue Mountains	0.97	-1.09	0.0011	10,920	58	<0.0001
4. Blue Ridge	0.89	0.12	0.0006	9,618	93	<0.0001

5. Cascades	0.96	-0.47	0.0008	77,688	132	<0.0001
6. Idaho Batholith	0.90	-1.85	0.0014	6,367	116	<0.0001
7. Klamath Mountains	0.97	-0.01	0.0006	1,882	43	<0.0001
8. Rockies	0.96	-1.25	0.0012	44,117	274	<0.0001
9. Sierra Nevada	0.96	-1.49	0.0013	91,413	182	<0.0001
10. Wasatch-Uinta Mountains	0.95	-2.50	0.0018	13,220	157	<0.0001

Table S3

Summary statistics of Discriminant Function Analyses (DFAs) predicting landscape membership into each of three climate vulnerability classes.

Mountain Range	DFA models using 80% as training and 20% as testing dataset		Coefficients of Linear Discriminants
	Accuracy (%)	p-value	
1. Appalachians	100	<0.0001	12.30
2. Arizona–New Mexico Mountains	95	<0.0001	10.90
3. Blue Mountains	96	<0.0001	8.35
4. Blue Ridge	96	<0.0001	21.94
5. Cascades	96	<0.0001	6.44
6. Idaho Batholith	99	<0.0001	10.87
7. Klamath Mountains	94	<0.0001	9.25
8. Rockies	99	<0.0001	6.81
9. Sierra Nevada	97	<0.0001	7.44
10. Wasatch-Uinta Mountains	98	<0.0001	8.30
